# Rethinking Senescence Hallmarks in the Brain: Lessons From Peripheral Tissues and Challenges in Defining Neuronal Senescence

**DOI:** 10.1111/acel.70719

**Published:** 2026-09-11

**Authors:** Miraj Ud Din Momand, Kristina Macova, Dominika Fricova

**Affiliations:** ^1^ Institute of Neuroimmunology Slovak Academy of Sciences Bratislava Slovakia; ^2^ The Unit for Translational Research of Neurodegenerative Diseases, 2nd Department of Neurology, Faculty of Medicine Comenius University Bratislava Slovakia; ^3^ Institute of Histology and Embryology, Faculty of Medicine Comenius University Bratislava Slovakia

**Keywords:** alpha‐synuclein, Alzheimer's disease, cellular senescence, markers, models, neurodegeneration, neuronal senescence, Parkinson's disease, tau

## Abstract

Cellular senescence is increasingly recognized as a fundamental driver of aging and age‐related diseases. Studies in peripheral tissues have revealed that senescence is not a uniform cellular state, but a highly heterogeneous phenotype shaped by distinct combinations of DNA damage, mitochondrial dysfunction, chromatin remodeling, impaired proteostasis, and diverse senescence‐associated secretory phenotype. This heterogeneity critically influences both the identification and therapeutic targeting of senescent cells. As senescent cells accumulate with age, they contribute to chronic low‐grade inflammation and tissue dysfunction, while their selective elimination can improve health span, establishing senescence as both a pathogenic mechanism and a therapeutic target in systemic aging. Emerging evidence now suggests that post‐mitotic neurons and other neural cell types can acquire senescence‐like states during aging and neurodegeneration. However, the direct application of canonical peripheral senescence markers to the nervous system remains conceptually and experimentally problematic, raising the risk of inconsistent or misleading interpretations across models and studies. In this review, we integrate insights from peripheral senescence biology to examine how cell type, inducing stressor, and experimental context shape senescence‐associated phenotypes across neural systems, including in vitro models, physiologically aged brains, and models of Alzheimer's and Parkinson's disease. By critically evaluating where canonical senescence hallmarks translate and where they fail to translate to post‐mitotic neurons, we propose that a refined, neuron‐centered framework is essential for moving the field beyond descriptive observations and for rigorously testing the causal contribution of senescent neural cells to neurodegeneration.

## Graphical abstract

Neuronal senescence‐like states arise from convergent intrinsic and disease‐associated stressors and are linked to persistent dysfunction, SASP‐like signaling, and altered glial interactions. Because no single marker is definitive in post‐mitotic neurons, multimarker and context‐dependent frameworks are required to define their role in neurodegeneration.

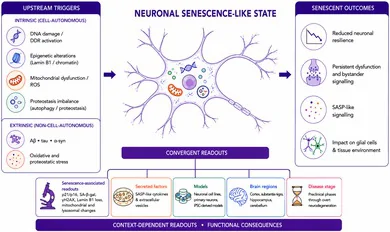

**Abbreviations**
AD: Alzheimer's disease
Aβ: amyloid beta
BBB: blood–brain barrier
CNS: central nervous system
DA: dopaminergic
DDR: DNA damage response
DIV: days in vitro
INS: induced neurons
iPSCs: induced pluripotent stem cells
LTC: long‐term cultivation
MPP+: 1‐methyl‐4‐phenylpyridinium
NDs: neurodegenerative diseases
NFTs: neurofibrillary tangles
NPCs: neural progenitor cells
p38 MAPK: p38 mitogen‐activated protein kinase
PD: Parkinson's disease
PHNs: primary rat hippocampal neurons
Rb: retinoblastoma protein
ROS: reactive oxygen species
SASP: senescence‐associated secretory phenotype
SATB1: special AT‐rich sequence‐binding protein 1
SA‐β‐gal: senescence‐associated β‐galactosidase
SNpc: substantia nigra pars compacta
TCDD: 2,3,7,8‐tetrachlorodibenzo‐p‐dioxin
TH: tyrosine hydroxylase
α‐syn: α‐synuclein
α‐syn PFFs: α‐syn pre‐formed fibrils

## 1 Introduction

Cellular senescence, classically characterized by irreversible cell cycle arrest and distinct phenotypic changes, plays a pivotal role in aging. Key markers associated with senescence include altered cell morphology, increased senescence‐associated β‐galactosidase (SA‐β‐gal) activity, DNA damage, chromatin reorganization, elevated levels of cyclin‐dependent kinase inhibitors such as p16^INK4a^ and p21^Waf1/Cip1^, upregulated anti‐apoptotic proteins such as Bcl‐2 and Bcl‐xL, enhanced production of reactive oxygen species (ROS), and the senescence‐associated secretory phenotype (SASP). SASP comprises increased secretion of inflammatory cytokines such as IL‐1 and IL‐6, growth factors such as vascular endothelial growth factor, and proteases such as matrix metalloproteinases (Coppe et al. 2010; Hernandez‐Segura et al. 2018; Herranz and Gil 2018).

Cellular senescence exemplifies antagonistic pleiotropy, a mechanism that offers advantages for survival and reproduction early in life but exerts detrimental effects in later stages of aging (Kirkwood and Austad 2000; Campisi 2013; Munoz‐Espin and Serrano 2014; Chinta et al. 2015; Borghesan et al. 2020). In youth, senescence contributes to tissue homeostasis by recruiting the immune system to eliminate damaged or potentially malignant cells. With advancing age, however, immune surveillance and clearance of senescent cells decline, leading to their accumulation and the establishment of a sustained pro‐inflammatory microenvironment that contributes to the onset and progression of age‐related pathologies such as Type 2 diabetes, cardiovascular disease, and cancer (Munoz‐Espin and Serrano 2014; Borghesan et al. 2020; He and Sharpless 2017). Animal studies demonstrating that removal of senescent cells can ameliorate disease phenotypes and extend lifespan underscore the therapeutic promise of selectively targeting these cells with senolytic drugs, which eliminate senescent cells while sparing their non‐senescent counterparts (Baker et al. 2011, 2016; Kirkland and Tchkonia 2020).

**FIGURE 1:**
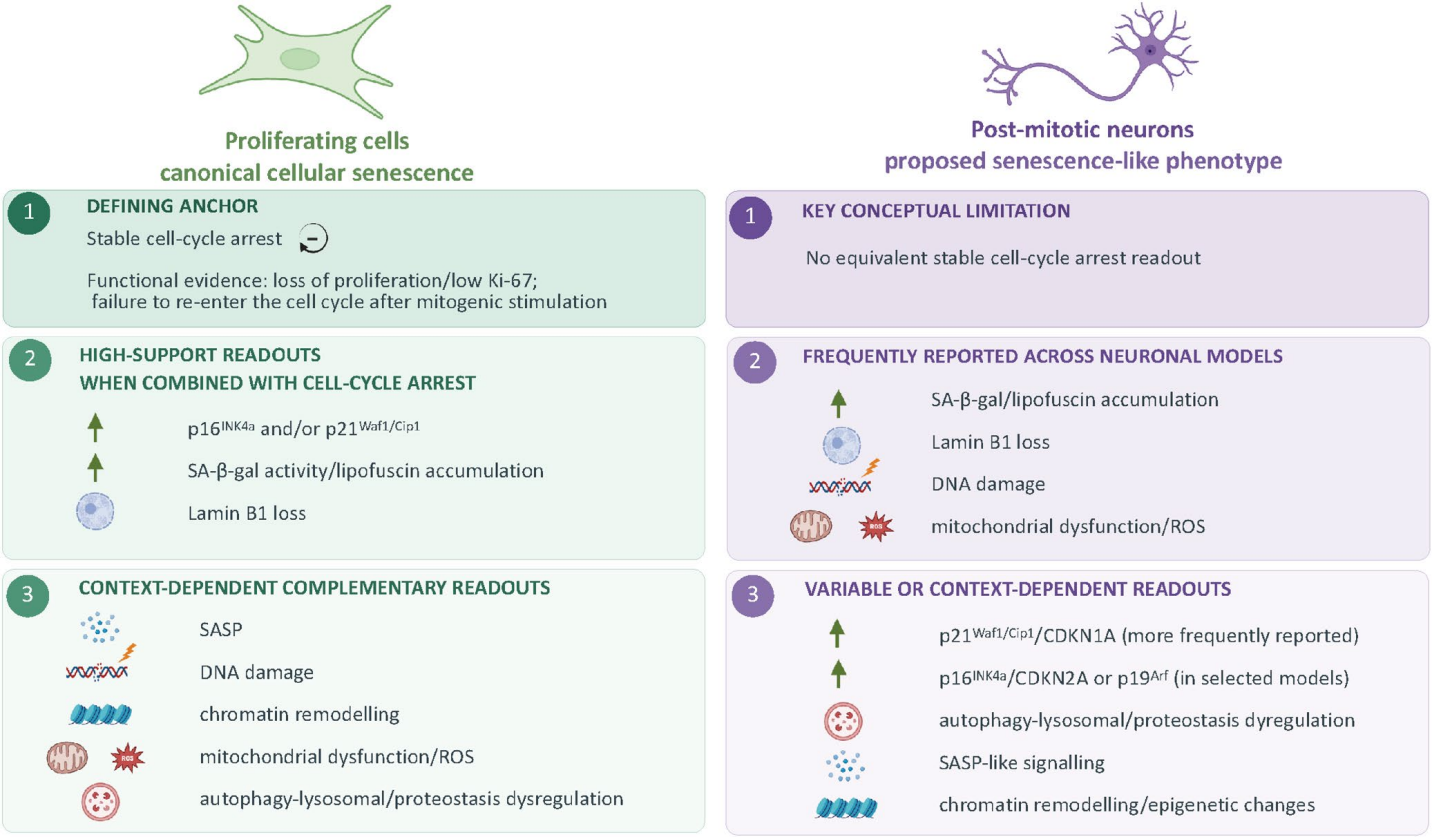
Hierarchies of senescence evidence in proliferating cells and post‐mitotic neurons. The schematic contrasts the hierarchy of senescence‐associated readouts in proliferating peripheral cells and post‐mitotic neurons. In proliferating cells, stable cell‐cycle arrest provides a defining anchor and is supported by complementary cell‐cycle, lysosomal, nuclear, metabolic, and SASP‐related markers. In neurons, no equivalent stable cell‐cycle arrest readout is available; instead, senescence‐like states are inferred from heterogeneous combinations of lysosomal/SA‐β‐gal changes, DNA damage, Lamin B1 loss, mitochondrial dysfunction, oxidative stress, and other context‐dependent features. p21^Waf1/Cip1^/CDKN1A is more frequently reported across neuronal models than p16^INK4a^/CDKN2A or p19^Arf^, whereas autophagy–lysosomal/proteostasis changes, SASP‐like signaling, and chromatin/epigenetic changes remain variable across models. The figure emphasizes the need for multimarker, context‐dependent interpretation of proposed neuronal senescence‐like states.

Studying cellular senescence in the nervous system is intrinsically challenging, as no single marker provides a definition that is simultaneously specific and universally applicable for senescent cells in brain tissue. Canonical senescence markers can also be induced by cellular stress, inflammation or differentiation, and their expression varies with cell type, senescence inducer and experimental context. Immunodetection of p16^INK4a^ in brain tissue is especially challenging, given its low baseline expression and variable antibody specificity, and such data should therefore be interpreted with caution. These issues are compounded by differences between experimental systems, including immortalized neuronal cell lines, primary neuronal cultures, mixed neuron–glia systems, animal models and human post‐mortem tissue, each of which captures distinct facets of stress and aging and can shape the resulting senescence‐associated phenotype. Reliable identification of senescence therefore requires integrating multiple complementary markers and critically evaluating the experimental system from which the data are derived (Hernandez‐Segura et al. 2018; Gorgoulis et al. 2019). The distinct evidence hierarchies for defining senescence in proliferating cells and identifying proposed senescence‐like states in post‐mitotic neurons are summarized in Figure 1.

In this review, we examine current evidence for senescence‐like states in neurons across physiological aging and neurodegenerative disease models, with particular emphasis on how differences in experimental systems, inducing stimuli, and marker selection shape the interpretation of neuronal senescence. By integrating insights from peripheral senescence biology with findings from the nervous system, we highlight the conceptual and methodological challenges of defining senescence in post‐mitotic neurons and discuss future directions for establishing refined, neuron‐centered senescence frameworks.

## 2 From Peripheral Tissues to the Central Nervous System

The prospect of therapeutically targeting senescent cells raises fundamental questions about how these cells are identified and how susceptible they are to senolytic interventions (Kirkland and Tchkonia 2020). Despite substantial progress in senescence research across physiological and pathological settings, most available data still come from mitotically active cells in peripheral tissues. As studies in the periphery have accumulated, they have revealed striking heterogeneity in the markers used to monitor senescent phenotypes and reinforced that the senescence response is highly context dependent (Table 1). The composition of this phenotype is shaped by the inducing stimulus, the intensity and duration of stress, cell type, stage of senescence, regulatory pathways, and the surrounding microenvironment, emphasizing the need to monitor and quantify multiple markers in parallel to reliably identify senescent cells (Coppe et al. 2010; Hernandez‐Segura et al. 2018; Herranz and Gil 2018; Kumari and Jat 2021).

**TABLE 1:** Variability of senescence marker profiles across studies of peripheral age‐related pathologies.

Pathology	Organism	Markers of senescence							References
		SA‐β‐gal	p16	ARF	p21	IL‐1	IL‐6	TNFa	
Osteoarthritis	Mouse	✔	✔		✔		✔		Xu et al. (2017)
	Mouse	✔	✔		✔	✔	✔		Jeon et al. (2017)
	Human	✔	✔		✔	✔	✔		
Osteoporosis	Mouse		✔		✔	✔	✔	✔	Farr et al. (2016)
Sarcopenia	Mouse	✔	✔	✔			✔		Sousa‐Victor et al. (2014)
Fibrotic pulmonary disease	Mouse		✔				✔	✔	Schafer et al. (2017)
	Human	✔	✔		✔	✔	✔		
Pulmonary dysfunction	Mouse		✔	✔	✔				Hashimoto et al. (2016)
Obstructive pulmonary disease	Human	✔	✔				✔	✔	Tsuji et al. (2010)
Insulin resistance	Mouse	✔	✔		✔		✔	✔	Aguayo‐Mazzucato et al. (2019)
	Human	✔	✔				✔		
Atherosclerosis	Mouse	✔	✔	✔		✔		✔	Childs et al. (2016)
Cardiac dysfunction	Mouse	✔	✔			✔	✔		Lewis‐McDougall et al. (2019)
	Human	✔	✔			✔	✔		
Kidney dysfunctions	Mouse	✔	✔	✔		✔	✔	✔	Baker et al. (2016)

*Note:* The table highlights the variability in the use of commonly assessed senescence markers across studies investigating peripheral age‐related pathologies. A check mark (✓) indicates that the corresponding marker was evaluated in the study. The absence of a check mark indicates that the marker was not assessed. p16 (p16^INK4a^); ARF (alternative reading frame protein p19^Arf^ in mice, p14^ARF^ in humans); p21 (p21^Waf1/Cip1^); SA‐β‐gal (senescence‐associated β‐galactosidase).

Building on insights from peripheral tissues, investigations of senescence in the central nervous system (CNS) have initially centered on mitotically active glial cells (Chinta et al. 2015, 2013, 2018; Bhat et al. 2012; Baker and Petersen 2018; Bussian et al. 2018; Kritsilis et al. 2018). Numerous studies have reported an increased presence of cells with senescence‐associated features in the mammalian brain during normal aging and in the course of chronic disorders such as amyotrophic lateral sclerosis, multiple sclerosis, Alzheimer's disease (AD), and Parkinson's disease (PD) (Chinta et al. 2015, 2013; Baker and Petersen 2018; Kritsilis et al. 2018; Wissler Gerdes et al. 2020; Martinez‐Cue and Rueda 2020; Papadopoulos et al. 2025). Glial cells appear particularly prone to both replicative and stress‐induced senescence, thereby compromising CNS homeostasis through functional impairment and SASP secretion (Chinta et al. 2015; Kritsilis et al. 2018; Evans et al. 2003). In combination with the age‐related loss of brain cells, this disruption can drive synaptic damage, loss of specific neuronal populations, white matter atrophy, and consequent cognitive and motor decline, including memory impairments and other features of CNS aging (Chinta et al. 2015; Baker and Petersen 2018; Kritsilis et al. 2018; Martinez‐Cue and Rueda 2020). Analogous to findings in peripheral tissues, selective elimination of senescent glia has been shown to ameliorate behavioral and cognitive deficits in animal models (Chinta et al. 2018; Bussian et al. 2018; Zhang et al. 2019). Although the precise etiology of many CNS disorders remains unresolved, these observations point to a potentially important contribution of senescence in brain cells. A key turning point came with the work of Jurk et al., who demonstrated that not only proliferative glial cells but also non‐dividing, terminally differentiated neurons can acquire a senescence‐like phenotype (Jurk et al. 2012).

## 3 Neuronal Senescence in Aging Models

### 3.1 Long‐Term Cultivation as an In Vitro Model of Aging

Long‐term cultivation (LTC) is an effective tool to simulate aging in vitro, particularly to induce senescence in dividing cells, such as fibroblasts and epithelial cells. Several studies focused on analyzing senescence in the nervous system pointed out that neurons, like other cell types, may undergo senescence‐like changes under LTC conditions (Moreno‐Blas et al. 2019; Ishikawa and Ishikawa 2020; Dong et al. 2011).

To decipher the molecular basis of neuronal senescence, Moreno‐Blas and colleagues employed an LTC model of senescence by using prenatal rat cortical cultures maintained for up to 40 days in vitro (DIV) (Moreno‐Blas et al. 2019). Neurons comprised 51% of the cultured cells at 26 DIV, while at 40 DIV, glia predominated (73%). Without the presence of external stimuli, these neurons started to exhibit senescence features, including increased SA‐β‐gal activity, lipofuscin accumulation, and altered nuclear morphology. However, the presence of neurons with high SA‐β‐gal activity but preserved nuclear morphology, underscored the limited reliability of SA‐β‐gal as a stand‐alone marker of neuronal senescence (Piechota et al. 2016). At 26 DIV, neurons but not glia showed increased expression of p21^Waf1/Cip1^ and γH2AX foci, suggesting that neurons might enter a senescent state before glia in this model, possibly stimulated by the DNA damage response (DDR) pathway. Impaired autophagy, a central cellular recycling pathway, was also evident, as reflected by the accumulation of LC3‐positive puncta together with increased p62 levels, collectively suggesting impaired autophagic degradation and reduced autophagic flux in long‐term cultured neurons. In parallel, SASP‐related alterations emerged, including intraneuronal accumulation of the SASP‐associated transcription factor GATA4 and selective changes in cytokine expression, most notably elevated MCP‐1 but not IL‐1α, IL‐1β, IL‐6, TNFα, or IFNγ, consistent with a distinct SASP profile. As these long‐term cortical cultures contained both neurons and astrocytes, the SASP profile measured in the conditioned medium likely reflected the combined contribution of both cell types and therefore should not be attributed solely to neurons without additional cell type‐specific analyses. Concerning the paracrine effects of SASP, conditioned media collected from these long‐term mixed cortical cultures induced senescence‐like changes in glial cells and fibroblasts, but not in early‐passage neurons, pointing to cell type‐ and age‐specific susceptibility to paracrine SASP effects (Moreno‐Blas et al. 2019).

Primary rat hippocampal neurons (PHNs) under LTC conditions (28 DIV) also exhibited senescence hallmarks, including SA‐β‐gal positivity, nuclear abnormality, Lamin B1 loss, chromatin reorganization, upregulated p16^INK4a^, activated p38 mitogen‐activated protein kinase (p38 MAPK), GATA4 accumulation, and increased SASP components such as CXCL‐1 compared to 7 DIV. Interestingly, these changes were observed without notable DNA double‐strand breaks or increased p21^Waf1/Cip1^ expression. PHNs exhibited proteostasis failure, characterized by accumulation of ubiquitinated proteins and amyloid beta (Aβ) aggregates, closely resembling features of aging and AD pathology, a pattern likewise reported in cortical neurons (Table 2). Autophagic flux analysis revealed active autophagy at early time points (7–14 DIV), which declined by 28 DIV as shown by reduced bafilomycin A1‐dependent p62 puncta, consistent with findings by Moreno‐Blas and colleagues (Moreno‐Blas et al. 2019). Rapamycin‐mediated mTOR pathway inhibition ameliorated senescence features in PHNs, suggesting the role of this pathway in maintaining neuronal health. Interestingly, at 28 DIV, PHNs displayed enhanced stress resilience, upregulated pro‐survival factor Bcl‐2, and decreased pro‐apoptotic Puma, indicating that neuronal senescence may serve as a protective, pro‐survival function under chronic stress conditions (Ishikawa and Ishikawa 2020). Together, these studies illustrate that neuronal senescence phenotypes vary substantially depending on neuronal subtype, culture duration, and the markers analyzed, emphasizing the difficulty of defining a unified senescence signature in post‐mitotic neurons.

**TABLE 2:** Senescence‐like phenotypes across neuronal models of aging and neurodegeneration.

Study/model	Biological system	Inducing context	Senescence‐related readouts used	Main phenotype reported	Interpretation	References
*A. In vitro and ex vivo neuronal models*						
Long‐term cultured cortical neurons/glia	Wistar rat cortical cultures	Long‐term cultivation	γH2AX, nuclear changes; p21; SA‐β‐gal, lipofuscin, autophagy impairment; MCP‐1	DNA damage, autophagy and SASP‐associated senescence features emerge during prolonged culture	Senescence‐like	Moreno‐Blas et al. (2019)
Long‐term cultured primary hippocampal neurons	E18 rat primary hippocampal neurons	Long‐term cultivation	Nuclear changes, Lamin B1 loss, chromatin changes; p16, p38 MAPK, GATA4; SA‐β‐gal; CXCL‐1/PAI‐1/IGFBP‐4/7	Proteostasis failure, SASP‐like changes and stress‐resilient neuronal senescence program	Senescence‐like	Ishikawa and Ishikawa (2020)
Long‐term cultured cortical neurons	E18 rat primary cortical neurons	Long‐term cultivation	Lamin B1 loss; p16, p38 MAPK; SA‐β‐gal, impaired autophagy; CXCL‐1/IGFBP‐2/4	Senescence‐associated changes with proteostasis and autophagy defects	Senescence‐like	Ishikawa and Ishikawa (2020)
Rotenone‐exposed neuronal cells	Differentiated SH‐SY5Y and primary cortical neurons	Rotenone ± α‐syn fibrils	p21, active Rb; SA‐β‐gal, impaired autophagy; ROS	LRRK2/p53‐p21‐linked senescence program associated with α‐syn accumulation	Senescence‐like	Ho et al. (2019)
SATB1‐deficient DA neurons	Human ESC‐derived DA neurons	SATB1 knockout	morphology; Lamin B1 loss; p21; SA‐β‐gal, autophagy defects, lipofuscin; IGFBP7; ROS	p21‐dependent senescence‐like phenotype with selective vulnerability of DA neurons	Senescence‐like	Riessland et al. (2019)
SATB1‐deficient cortical neurons	Human embryonic stem cells‐derived cortical neurons	SATB1 knockout	Minimal senescence readouts detected	Cortical neurons lacked a comparable phenotype	Negative/cell‐type‐specific	Riessland et al. (2019)
MPP + ‐treated DA neurons	Rat N27 DA cells	MPP+	Lamin B1 loss; p16, p21	Early senescence‐like changes precede overt degeneration	Partial/senescence‐like	Verma et al. (2021)
α‐syn PFF‐treated neurons	Rat N27 DA neurons and primary cortical neurons	α‐syn PFFs	Lamin B1 loss; SATB1, p16, p21	Stronger glial than neuronal senescence response; neuronal changes are limited or transient	Mixed/context‐dependent	Verma et al. (2021)
Aβ42‐treated neural progenitors	Adult mouse hippocampal NSPCs	Oligomeric Aβ42	morphology; p16INK4a, Rb, p38 MAPK; SA‐β‐gal; ROS	Senescence‐associated impairment of proliferation and differentiation	Senescence‐like	He et al. (2013)
AD patient‐derived INs	Human fibroblast‐derived cortical iNs	Aging/AD pathology	nuclear changes; CDKN2A; SA‐β‐gal; SASP factors, inflammatory genes; epigenomic changes	Age‐ and neuron‐specific senescence‐like signature absent from rejuvenated iPSC‐neurons	Senescence‐like	Herdy et al. (2022)
α‐syn overexpression model	Differentiated SH‐SY5Y cells	Ectopic α‐syn expression	H3K9me3, γH2AX; p21; SA‐β‐gal; mitochondrial abnormalities	DNA damage response‐associated senescence‐like phenotype in α‐synucleinopathy model	Senescence‐like	Yoon et al. (2022)
A53T α‐syn + iron overload	Differentiated rat PC12 cells	A53T α‐syn + iron overload	p16, p21; SA‐β‐gal; ROS	Iron‐dependent amplification of α‐syn‐associated senescence phenotype	Senescence‐like	Shen et al. (2024)
*B. In vivo and human tissue studies*						
Physiological brain aging	Aged C57BL/6 Purkinje and cortical neurons	Natural aging	γH2AX, macroH2A; p21, p38 MAPK; SA‐β‐gal, lipofuscin; IL‐6; ROS	Post‐mitotic neurons acquire pro‐inflammatory, DNA damage‐associated senescence features with age	Senescence‐like	Jurk et al. (2012)
APP/PS1 hippocampal progenitors	APP/PS1 mice, dentate gyrus NSPCs	Aβ pathology	p16, SA‐β‐gal, FPR2‐related signaling	Aβ‐associated progenitor senescence linked to impaired neurogenesis	Senescence‐like	He et al. (2013)
5XFAD hippocampal neurons	5XFAD mouse hippocampus	Aging/Aβ pathology	p16, p21	Early p16 increase associated with worsening cognition, but incomplete classical senescence profile	Partial/suggestive	Wei et al. (2016)
TauNFT mice	rTg(tauP301L)4510 mouse hippocampus	Tau aggregation/NFTs	γH2AX; CDKN2A, CDKN1A; SASP factors; mitochondrial dysfunction	NFT‐linked senescence program associated with brain degeneration and responsive to senolytics	Senescence‐associated/strong evidence	Musi et al. (2018)
Human AD cortex/CSF	Post‐mortem AD brain and CSF	AD pathology	CDKN2A, p16; SASP	Senescent‐like neuronal clusters and SASP‐associated proteins in AD tissue and CSF	Senescence‐associated	Herdy et al. (2022)
Human cortical excitatory neurons	Post‐mortem AD brains	Tau/NFT pathology	Morphology; Lamin B1 loss; p19; lipofuscin	Senescent‐like excitatory neurons spatially associated with NFTs	Senescence‐associated	Dehkordi et al. (2021)
SATB1‐deficient DA neurons in vivo	Mouse midbrain DA neurons	SATB1 knockdown	CDKN1A; lipofuscin; SASP factors	p21‐linked neuronal senescence‐like state with neuroinflammatory component	Senescence‐like	Riessland et al. (2019)
α‐syn PFF model	Mouse midbrain and human PD midbrain	α‐syn PFF pathology/PD pathology	Lamin B1 loss; SATB1, p21, p16	Predominantly glial senescence signature with more indirect neuronal involvement	Mixed/glia‐dominant	Verma et al. (2021)
A53T α‐syn overexpression	Mouse SNpc/motor cortex/hippocampus	A53T α‐syn overexpression	γH2AX; p16, p21; SA‐β‐gal, lipofuscin; IL‐4/5/6; oxidative stress	Early senescence‐like features precede overt DA neuron loss and motor dysfunction	Senescence‐like/early pathogenic event	Shen et al. (2024), Yoon et al. (2022)

*Note:* Studies are grouped according to experimental context and biological system. Senescence‐related readouts are organized into functional categories including nuclear/DNA‐associated alterations, cell‐cycle regulators, lysosomal/proteostasis‐associated markers, SASP‐associated factors, and other senescence‐related changes. The table illustrates the substantial variability in marker selection and phenotype composition across neuronal models, highlighting the lack of a unified neuronal senescence signature.: 2,3,7,8‐tetrachlorodibenzodioxin (TCDD), 1‐methyl‐4‐phenylpyridinium (MPP+), α‐synuclein (α‐syn), Alzheimer's disease (AD), amyloid beta (Aβ), cerebrospinal fluid (CSF), dopaminergic (DA), induced neurons (INs), neural stem/progenitor cells (NSPCs), neurofibrillary tangles (NFTs), pre‐formed fibrils (PFFs), reactive oxygen species (ROS), substantia nigra pars compacta (SNpc), p16^INK4a^ (p16), p21^Waf1/Cip1^ (p21).

### 3.2 Evidence From Physiological Brain Aging: Natural Aging in Mice

In 2012, Jurk and colleagues presented a discovery that challenged the conventional understanding of cellular senescence, extending the scope of research to post‐mitotic cells (Jurk et al. 2012). Their investigation, primarily conducted on a widely utilized inbred C57BL/6 mouse strain, unveiled the emergence of a senescence‐like phenotype in various neuronal subtypes during aging. Cerebellar sections from aged mice exhibited clear signs of senescence, including elevated SA‐β‐gal activity, γH2AX, activated p38 MAPK, IL‐6, and lipofuscin accumulation. Purkinje neurons from 32‐month‐old mice displayed increased DNA damage, p38 MAPK activation, accumulation of 4‐hydroxynonenal, a marker of lipid peroxidation, and higher IL‐6 and lipofuscin levels compared to 4‐month‐old mice. Similarly, aged cortical neurons exhibited senescence‐associated changes, marked in part by increased deposition of the histone variant macroH2A in heterochromatin, a hallmark of chromatin remodeling during senescence. Jurk and colleagues further explored the role of p21^Waf1^ ^/Cip1^ and telomerase activity in neuronal senescence in aged mice. While the knock‐out of CDKN1A, the gene encoding p21^Waf1^ ^/Cip1^, reduced several senescence‐associated markers, only levels of 4‐hydroxynonenal changed significantly compared with wild‐type mice. In contrast, loss of telomerase caused telomere dysfunction, stronger DDR and higher IL‐6 levels, activation of p38 MAPK, and increased oxidative stress in Purkinje and cortical neurons. Notably, double knockout mice in which both CDKN1A and the telomerase gene were deleted revealed that these effects depended on p21^Waf1^ ^/Cip1^ signaling downstream of DDR activation. Interestingly, the introduction of mild dietary restrictions reduced senescence‐related markers in Purkinje cells. Taken together, the data support a model in which physiological aging promotes DNA damage and senescence‐like, pro‐inflammatory neuronal states that can be partly alleviated by dietary intervention (Jurk et al. 2012).

Importantly, direct comparison between physiological aging and long‐term culture models remains limited by differences in marker panels analyzed, underscoring the need for more standardized, multi‐parametric approaches to define neuronal senescence. A comparison of senescence‐associated readouts across neuronal aging models is summarized in Table 2.

## 4 Neuronal Senescence and Neurodegeneration

In‐depth senescence investigations in the nervous system are particularly attractive for the research of age‐related neurodegenerative diseases (NDs), as they would enhance our understanding of disease‐related mechanisms and could serve as a new therapeutic avenue. NDs represent a spectrum of heterogeneous and progressive pathologies characterized by the gradual loss of selectively vulnerable neuronal populations. These include amyloidosis (e.g., AD), tauopathies (e.g., AD, progressive supranuclear palsy), α‐synucleinopathies (e.g., PD), and transactivation response DNA binding protein 43 proteinopathies (e.g., amyotrophic lateral sclerosis) (Dugger and Dickson 2017). The rising prevalence of NDs worldwide is intrinsically linked to the aging demographics of the global population. Despite distinct etiological factors and clinical manifestations, NDs share fundamental features, including the increase in intracellular oxidative stress and neuroinflammation, which intriguingly parallel the traits associated with cellular senescence (Kritsilis et al. 2018; Martinez‐Cue and Rueda 2020; Dugger and Dickson 2017; Maciel‐Baron et al. 2018). While the role of different cell types, including post‐mitotic neurons, in the landscape of neurodegeneration is yet to be fully deciphered, mounting evidence of neurons exhibiting senescence‐like features in patients and experimental models of various NDs implies their involvement in the pathological processes (Nicaise et al. 2019; Dehkordi et al. 2021; Hu et al. 2022).

### 4.1 Neuronal Senescence in Toxin‐Based Models of Neurodegeneration

Whereas physiological aging demonstrates that neurons can gradually acquire senescence‐like features in the absence of overt pathology, experimental models of neurodegeneration provide an opportunity to investigate how disease‐associated stressors accelerate or reshape these processes. Among these, toxin‐based models have been widely used to examine whether oxidative stress, mitochondrial dysfunction and proteostatic impairment are sufficient to induce neuronal senescence‐like phenotypes relevant to neurodegenerative disease.

Rotenone, a naturally occurring pesticide linked to PD pathology, has been used to investigate whether mitochondrial stress and LRRK2 activation promote senescence‐like changes in neuronal cells. Chronic low‐dose exposure to rotenone has been shown to upregulate leucine‐rich repeat kinase 2 (LRRK2), a multifunctional enzyme involved in synaptic transmission, membrane trafficking, cytoskeletal remodeling, and phosphorylation of various substrates (e.g., β‐tubulin, tau) (Jeong and Lee 2020; Ho et al. 2019). Importantly, mutations in the LRRK2 gene play a critical role in the pathogenesis of familial and sporadic PD (Ito and Utsunomiya‐Tate 2023). Building upon previous observations, Ho et al. examined whether rotenone induces PD‐like pathology by promoting senescence induction in neurons. Human dopaminergic (DA) neuron‐like cells (differentiated SH‐SY5Y cells) were treated with rotenone (1 μM) with or without the LRRK2 kinase inhibitor GSK2578215A for 48 h. The treatment resulted in increased expression and activation of LRRK2, elevated SA‐β‐gal, increased synthesis of p53 and p21^Waf1/Cip1^, reduced levels of phosphorylated Rb, a compromised autophagy‐lysosomal pathway, higher ROS production, and enhanced accumulation of a major protein associated with PD pathology, α‐synuclein (α‐syn). The relationship between senescence‐like changes and α‐syn aggregation was examined using α‐syn fibrils (70 nM) added to the media. Intriguingly, co‐treatment with GSK2578215A alongside rotenone mitigated all these features, suggesting a potential therapeutic approach. These findings were replicated in primary rat cortical neurons, with no significant change in p16^INK4a^ expression, reinforcing that rotenone‐induced senescence primarily operates via p53‐p21^Waf1/Cip1^ axis driven by LRRK2 kinase activity and autophagy impairment, leading to α‐syn accumulation (Ho et al. 2019). These findings add to growing evidence implicating LRRK2 in neuronal senescence and PD progression (Ho et al. 2021). The effect of LRRK2 kinase inhibition on rotenone‐induced senescence was further explored in C57BL/6J mouse midbrain, subjected to intraperitoneal injections with rotenone (0.75 mg/kg body weight) and MLi‐2 (1 mg/kg body weight), a blood–brain barrier permeable LRRK2 kinase inhibitor, once every second day for 2 weeks. The results indicated a modest, nonsignificant decrease in locomotor activity in rotenone‐treated mice, as assessed by the rotarod test, which measures motor coordination and balance. MLi‐2 non‐significantly mitigated the rotenone‐induced activation of the LRRK2 kinase and p53–p21^Waf1/Cip1^ pathway in the midbrain. Notably, rotenone administration led to a marked increase in SA‐β‐gal activity, which was significantly decreased by MLi‐2, and increased fibrillar α‐syn oligomer accumulation, which was slightly attenuated upon co‐treatment with MLi‐2 (Ho et al. 2019). Rotenone thus appears to promote cellular senescence and α‐syn aggregation primarily through upregulation of LRRK2 activity, suggesting that pharmacological inhibition of this kinase may offer a potential therapeutic strategy. At the same time, the relatively modest effects observed in vivo, compared with the more pronounced in vitro responses, underscore the complexity of translating molecular findings into effective interventions in intact organisms and emphasize the need for further studies to close this gap.

Finally, 1‐methyl‐4‐phenylpyridinium (MPP+), a neurotoxin, is commonly used in cellular models of PD for its selective toxicity toward DA neurons in the substantia nigra pars compacta (SNpc). In rat DA N27 cells, treatment with 640 μM MPP+ for 24 h decreased Lamin B1, and increased p16^INK4a^ and p21^Waf1/Cip1^ (Verma et al. 2021). These results partially support previous results, where fetal rat DA neurons treated with 5 μM MPP^+^ showed nuclear changes despite preserved cell bodies, indicating that many cells remained viable at 24 h (Nakamura et al. 2000). Together, these findings imply that MPP+ could induce senescence in cultured DA neurons before they degenerate due to excessive neurotoxic stress (Verma et al. 2021; Nakamura et al. 2000).

Collectively, toxin‐based models reproduce selected features of neuronal senescence, yet the specific combination of markers differs substantially between stressors, reinforcing the notion that neuronal senescence cannot be defined by a single universal marker set. Importantly, much of the evidence in this area derives from immortalized neuronal or neuroblastoma‐derived cell lines, including SH‐SY5Y, N27 and PC12 cells, often complemented by primary rodent neuronal cultures. Although these systems have been instrumental in identifying pathways associated with senescence‐like responses, their immortalized origin, ongoing proliferation and incomplete resemblance to mature post‐mitotic neurons impose important limitations when extrapolating findings to neuronal aging in vivo. Accordingly, SA‐β‐gal, p16^INK4a^ and p21^Waf1/Cip1^ readouts in these cell lines may capture stress and cell‐cycle responses rather than bona fide neuronal senescence. Primary neuronal cultures provide a more physiological complement, but their neuronal and glial composition should likewise be considered unless it is explicitly defined.

### 4.2 Neuronal Senescence in Age‐Related Neurodegenerative Diseases

Although considerable research has focused on unveiling the initial causes of NDs, early and specific pathogenetic mechanisms remain elusive. This knowledge gap significantly hinders the development of effective therapeutic interventions. Current strategies targeting protein aggregates in conditions like AD and PD have shown limited success, prompting the exploration of alternative approaches. Given the role of senescence in aging and health‐deteriorating conditions, understanding its connection to NDs has gained significant attention. Research on senescent neurons holds promise in uncovering the underlying mechanisms of NDs and may open new avenues for developing targeted therapies. This complexity is further reflected in studies of AD and PD, where senescence‐associated phenotypes vary considerably depending on disease stage, model system, and the markers selected for analysis.

#### 4.2.1 Senescence in AD

AD is currently the most common cause of dementia in older adults, accompanied by cognitive and behavioral impairments. On the molecular level, AD is characterized by extracellular amyloid plaques, consisting predominantly of amyloid beta (Aβ) protein, and neurofibrillary tangles (NFTs), comprised mainly of hyperphosphorylated tau. The accumulation of these aggregates leads to disrupted cellular functions, promoting neuronal and synaptic loss, which contributes to chronic inflammation, neurodegeneration, and cognitive decline (Baker and Petersen 2018; Kritsilis et al. 2018; Martinez‐Cue and Rueda 2020; Crews and Masliah 2010; Musi et al. 2018). Pathological processes leading to AD begin years prior to noticeable symptoms, and some senescence‐related events have been proposed to be critically involved. For example, oxidative stress, inflammation, or mitochondrial dysfunction can be observed in patients and mouse models of AD from early stages preceding the visible amyloid plaques and NFTs deposition (Bussian et al. 2018; Kritsilis et al. 2018; Martinez‐Cue and Rueda 2020).

##### 4.2.1.1 Cell‐Based Models of AD and Neuronal Senescent Phenotypes

Several studies highlighted the involvement of Aβ and NFTs in inducing premature senescence in cultured brain cells. Moreover, their formation was shown to increase with the duration of the senescence response (Bhat et al. 2012; Bussian et al. 2018; Martinez‐Cue and Rueda 2020; Wei et al. 2016). In neural cells, oligomeric Aβ1–42 (Aβ42), which is considered to be the most toxic form of Aβ, was shown to support pathological processes associated with AD by inducing senescence. In vitro studies demonstrated that Aβ42 exerts concentration‐ and time‐dependent effects on proliferation, differentiation, apoptosis, and senescence in primary hippocampal neural progenitor cells (NPCs) isolated from 8‐week‐old male C57BL/6 mice. High Aβ42 concentrations (10 μM) increased apoptosis, resulting in smaller and fewer neurospheres with altered morphology. Lower concentrations (2.5–5 μM) of Aβ42 reduced the cells' capacity to proliferate and differentiate while increasing their resistance to apoptosis, consistent with the induction of a senescent state. Expanding their analysis, the authors treated NPCs with Aβ42 (2.5–10 μM) for up to 5 days, observing a concentration‐dependent rise in senescence‐associated markers with the strongest effect at 10 μM. These findings point to a mixed population of senescent and apoptotic NPCs, potentially influencing the obtained read‐outs. Senescent‐like NPCs exhibited reduced neurosphere sizes, shortened processes, and reduced expression of neural phenotype markers β‐III‐tubulin and GFAP, indicating impaired neurogenesis and differentiation. Elevated SA‐β‐gal activity, changes in CDKN2A expression and reduced Rb phosphorylation, together with altered proliferation and survival, reinforce the notion that Aβ42 initiates a senescence‐like response in NPCs. Consistently, higher numbers of NPCs with senescence‐associated features were detected in the dentate gyrus of APP/PS1 transgenic mice, which co‐express amyloid precursor protein bearing the Swedish mutation (APPswe) and mutant human presenilin‐1, compared with wild‐type mice. Mechanistically, Aβ42 appeared to act via the formylpeptide receptor 2 (FPR2) and activation of the ROS–p38 MAPK signaling pathway, providing crucial insights into the molecular drivers of progenitor cell senescence in AD (He et al. 2013).

To investigate neuronal senescence in AD, Herdy and colleagues collected fibroblasts from 16 ad patients (13 sporadic and 3 familial) and 19 non‐demented controls and directly converted them into induced neurons (iNs). iNs retain age‐ and disease‐associated signatures, unlike neurons derived from induced pluripotent stem cells (iPSCs), which undergo epigenetic rejuvenation, making them a particularly suitable model for studying age‐related neurodegenerative diseases. AD iNs showed a significant upregulation of senescence‐ and inflammation‐related genes such as CDKN2A, MMP3, CXCL5/8, and IL‐6, accompanied by downregulation of several genes with neuron‐specific functions. This pattern indicates a senescence‐like response that is distinct from that seen in proliferating cell types and in rejuvenated neuron models such as iPSC‐derived neurons, highlighting a neuron‐ and age‐specific senescence signature in the context of AD (Herdy et al. 2022). Epigenomic analysis showed that the transcription start sites of senescence‐related genes were more accessible for transcription factors in AD iNs than in controls, with a positive correlation between CpG promoter methylation and gene expression, suggesting dysregulated epigenetic control. Transcription factor ETS1, known to regulate the expression of p16^INK4a^ and bind to hypermethylated promoters, was implicated in driving gene expression despite increased promoter methylation in AD neurons, indicating neuronal senescence, which was further supported by SA‐β‐gal staining. These changes were specific to aged neurons and were not present in rejuvenated iPSC‐derived neurons. Conditioned media from AD iNs contained SASP factors that activated astrocytes, inducing gene expression patterns characteristic of reactive astrogliosis and senescence, mirroring changes observed in AD brain tissue, including loss of glutamate transporters and synaptic genes. Interestingly, treatment with senolytics (dasatinib and quercetin) reduced the proportion of senescent neurons to control levels. Together, these findings suggest that senescent AD neurons can trigger astrocyte reactivity and contribute to neurodegenerative pathology, highlighting senescent neurons as a potential therapeutic target in AD and related disorders (Herdy et al. 2022).

##### 4.2.1.2 Mouse Models and Patients With AD and Neuronal Senescence Readouts

The interplay between AD‐related Aβ aggregation and neuronal senescence, previously shown in vitro, was further analyzed in APP/PS1 transgenic mice described above. In this model, Aβ accumulation was associated with impaired neuronal survival and neurogenesis (He, Jin et al. 2013). Immunohistochemistry confirmed Aβ deposits in the cerebral cortex and hippocampus of APP/PS1 mice but not in wild‐type controls. The number of SA‐β‐gal‐positive cells in transgenic mice increased by approximately 1.7‐fold in the hippocampus, specifically in the dentate gyrus, compared to controls. To explore the mechanism of Aβ‐induced senescence, brain sections were stained for FPR2, revealing a significant increase in FPR2‐positive cells in the dentate gyrus of APP/PS1 mice, supporting prior in vitro findings. Together, these observations indicate that accumulation of Aβ can trigger a senescence‐like program in hippocampal neural progenitor cells, contributing to neurogenesis dysfunction. Therefore, targeting FPR2 may offer a novel therapeutic approach to modulate neurogenesis in AD (He et al. 2013).

The impact of Aβ accumulation and neuronal senescence on cognition was examined in the 5XFAD mouse model, which develops Aβ plaques, gliosis, neuronal loss, and cognitive deficits recapitulating key aspects of AD, though lacking NFTs (Wei et al. 2016; Oblak et al. 2021). In the hippocampus of 5XFAD mice, expression of CDKN2A was markedly increased from approximately 7 months of age, while age‐matched wild‐type controls showed slight changes even in older ages. Interestingly, p53 or p21^Waf1/Cip1^ were upregulated only later in life (18 months). In line with these gene expression findings, increased CDKN2A/p16^INK4a^ pathway activation has been reported in the hippocampus of 5XFAD mice, suggesting that senescence‐associated mechanisms may be engaged relatively early during AD‐like pathology. Behavioral tests (Y‐maze, Morris water maze) confirmed cognitive impairments in 5XFAD mice versus controls. Follow‐up in vitro experiments showed a positive correlation between p16^INK4a^ expression and Aβ deposition, suggesting that Aβ may engage the CDKN2A/p16^INK4a^ pathway. Although CDKN2A/p16^INK4a^ expression increases in the brain during aging, this increase appears further exacerbated in the 5XFAD model and is reported to negatively correlate with cognitive performance (Wei et al. 2016).

To investigate the relationship between intraneuronal tau pathology and senescence in vivo, gene expression analyses were performed in rTg(tauP301L) 4510 (tauNFT) transgenic mice, a model exhibiting robust tau accumulation, neurodegeneration, and cognitive deficits (Musi et al. 2018). Gene expression analyses were performed on hippocampi obtained from tau NFT mice before (~2‐month‐old) and after (~6‐month‐old), to determine whether NFT‐containing neurons display a senescence‐like phenotype. Tau NFT mice displayed elevated levels of histone γH2AX, CDKN2A, CDKN1A, and two to 13‐fold higher expression of SASP‐associated factors such as TLR4, TNFα, CXCL1, and IL‐1β. Senescence‐related mitochondrial dysfunction was specifically observed in brain regions with persistent tau pathology and was absent in unaffected areas like the cerebellum. SA‐β‐gal staining revealed only sparse positive cells, including in very young mice (1 month), with signal correlating with brain size rather than pathology, suggesting limited utility in this model. Genetic ablation of the tau‐encoding gene MAPT reduced NFT burden, senescence marker expression, brain atrophy, and SASP production. Furthermore, analysis in tau transgenic 3xTgAD mice, which develop both Aβ and tau pathology, showed that upregulation occurred in parallel with NFT formation and correlated with NFT burden, but not with general protein accumulation or pre‐NFT tau species. Higher CDKN2A expression also strongly predicted brain atrophy. Finally, senolytic treatment with dasatinib and quercetin increased neuron‐specific proteins, enhanced cerebral blood flow, and reduced neurodegeneration, supporting the link between NFTs and CDKN2A‐mediated senescence and the potential therapeutic value of senescent cell clearance in tau‐driven neurodegeneration (Musi et al. 2018).

Post‐mortem analysis of AD brains revealed increased CDKN2A expression in the neuron‐rich prefrontal cortex, but not in the glia‐dominant frontal white matter. Subsequent analysis of samples collected from AD patients and controls revealed increased levels of SASP‐associated proteins, such as MMP‐10, CXCL‐5/6/9, CCL‐23/11, CST‐5, and IL‐7, which could be potentially released by senescent neurons (Herdy et al. 2022). Interestingly, three of the identified proteins elevated in cerebrospinal fluid of AD patients (MMP‐10, STAMBP, and EIF4EBP1) have previously been identified as AD biomarkers (Herdy et al. 2022; Whelan et al. 2019).

The association between NFTs and neuronal senescence in the context of AD was further investigated in a comprehensive study involving 76 postmortem human brains at various stages of the disease. Remarkably, the study revealed that more than 97% of cells exhibiting a senescence‐like phenotype, characterized by altered morphology, lipofuscin accumulation, and increased expression of p19, represented excitatory neurons. Furthermore, these senescent‐like neurons spatially coincided with the population bearing NFTs, suggesting a direct linkage between tau pathology and the induction of a senescence‐like state (Dehkordi et al. 2021).

Importantly, the senescence‐associated changes reported across AD models remain highly heterogeneous, with inconsistent detection and localization of canonical markers such as SA‐β‐gal, p16^INK4a^ and p21^Waf1/Cip1^, and with several studies relying on p16^INK4a^ immunostaining in brain tissue that is technically challenging due to low baseline expression and potential antibody‐specific background. For these reasons, in the present review, evidence based primarily on such p16^INK4a^ staining is interpreted cautiously and the emphasis is placed on multimarker readouts and convergent findings from gene expression, mitochondrial dysfunction, DNA damage and SASP‐associated signaling rather than on single‐marker IHC data. Together, this heterogeneity highlights the limitations of directly extrapolating peripheral senescence criteria to neurons and underscores the need for refined, neuron‐centred senescence frameworks.

Overall, the available data across cell‐based systems, mouse models and post‐mortem AD brains converge on the view that Aβ aggregation and tau pathology are consistently associated with senescence‐like changes in neural progenitors and excitatory neurons, including increased CDKN2A expression, DNA damage, mitochondrial dysfunction and SASP‐related signaling, and that these changes correlate with impaired neurogenesis, brain atrophy and cognitive decline. At the same time, the composition of the senescence‐like phenotype and the robustness of canonical markers such as SA‐β‐gal, p16^INK4a^ and p21^Waf1/Cip1^ vary markedly across models and brain regions, leaving open whether neuronal senescence represents a primary driver of AD pathology or a context‐dependent stress response that accompanies Aβ and tau accumulation.

#### 4.2.2 Senescence in PD

PD is currently the second most prevalent ND associated with motor, behavioral, and cognitive dysfunctions. At a histopathological level, PD is characterized by the loss of DA neurons in SNpc along with increased α‐syn aggregates called Lewy bodies. The link between senescence and PD was made when multiple studies identified increased senescence‐like traits in the cerebrospinal fluid, serum, astrocytes, and neurons of patients as well as cell‐based and mouse models of PD (Chinta et al. 2018; Baker and Petersen 2018; Kritsilis et al. 2018; Wissler Gerdes et al. 2020; Verma et al. 2021; Shen et al. 2024).

##### 4.2.2.1 Cell‐Based Models of PD and Neuronal Senescent Phenotypes

Mutations in several genes (e.g., SNCA or LRRK2) are known risk factors for developing PD. Recent studies have identified Special AT‐Rich Sequence‐Binding Protein 1 (SATB1), a DNA‐binding protein implicated in the organization of chromatin, as another PD‐linked genetic factor. While SATB1 appears to minimally affect gene expression in early DA neurons, it exerts greater influence on mature DA neurons, acting predominantly as a gene repressor (Riessland et al. 2019). SATB1 depletion in DA neurons in vitro led to senescence‐like features, including increased SA‐β‐gal activity, altered nuclear morphology, reduced Lamin B1, elevated p21^Waf1/Cip1^, lysosomal dysfunction, proteostasis defects, ROS accumulation, and mitochondrial damage, though without exhibiting signs of DNA damage or p16^INK4a^ upregulation. Senolytic drugs (azithromycin, fisetin, dasatinib/quercetin) selectively reduced viability in SATB1‐deficient DA neurons, whereas ABT‐737 was toxic to both knockout and control cells. This suggests that first‐line senolytics target senescence‐associated pro‐survival pathways in SATB1‐deficient neurons, while the Bcl‐2 family inhibitor ABT‐737 more broadly disrupts apoptotic regulation irrespective of the senescent‐like state. Mechanistically, SATB1 binds the CDKN1A regulatory region and suppresses p21^Waf1/Cip1^ expression. Its depletion enhances p21^Waf1/Cip1^ levels and senescence, which can be mitigated by p21^Waf1/Cip1^ downregulation. Notably, SATB1 loss did not induce senescence in cortical neurons, underscoring its cell‐type‐specific role (Riessland et al. 2019).

To investigate whether α‐syn pathology induces cellular senescence, Verma et al. treated various CNS cell types with α‐syn pre‐formed fibrils (PFFs). In rat N27 DA neurons, exposure to 1 μg/mL of α‐syn PFFs for up to 48 h reduced Lamin B1 and SATB1 levels, transiently upregulated p21^Waf1/Cip1^ at 12 h, which declined by 48 h, and unexpectedly reduced p16^INK4a^. In primary rat cortical neurons, exposure to 5 μg/mL α‐syn PFFs increased Lamin B1 and SATB1 expression at various time points, p21^Waf1/Cip1^ increased up to 48 h but declined by 72 h, while p16^INK4a^ levels remained unchanged. However, a gradual reduction in β‐III‐tubulin levels was observed, suggesting that neurodegeneration persisted beyond 48 h. The 3‐day treatment with α‐syn PFFs also induced senescence in primary glia. In astrocytes, α‐syn PFFs treatment reduced Lamin B1, SATB1, and p16^INK4a^ levels, alongside an increase in p21^Waf1/Cip1^ and GFAP levels, indicating senescence activation. Microglia responded to α‐syn PFFs by a gradual decrease in Lamin B1 and p16^INK4a^, while the levels of p21^Waf1/Cip1^ and Iba‐1 increased, also indicating senescence activation (Verma et al. 2021). These findings highlight the complex and heterogeneous nature of senescence response, significantly influenced by the concentration and exposure time of inducing stimuli as well as the affected cell type.

Research by Yoon et al. employing ectopic expression of α‐syn from recombinant adenoviral vectors in the SH‐SY5Y further confirmed the relationship between α‐syn and cellular senescence (Yoon et al. 2022). Transcriptomic and proteomic analyses revealed early upregulation of p53 and p21^Waf1/Cip1^ peaking at 48 h post α‐syn expression, consistent with their transient role in senescence initiation (He and Sharpless 2017; Kumari and Jat 2021). α‐Syn overexpression led to an increase in SA‐β‐gal–positive cells, elevated levels of histone H3K9 trimethylation, a hallmark of senescence‐associated heterochromatin, and the emergence of mitochondrial abnormalities. The data also revealed a parallel increase in γH2AX signal and DDR activation with α‐syn levels, suggesting a potential link between α‐syn‐induced stress response and DNA damage. The study proposed that α‐syn may predominantly influence the formation of double‐stranded DNA breaks and non‐homologous end‐joining repair mechanisms, contributing to the induction of senescence in SH‐SY5Y cells (Yoon et al. 2022).

Previous reports highlighted the synergy between α‐syn and iron overload in PD pathogenesis. In PC12 cells with stable overexpression of the A53T α‐syn mutant, which is linked to early‐onset PD, senescence markers such as SA‐β‐gal, p16^INK4a^ and p21^Waf1/Cip1^ were significantly upregulated. These levels further increased, accompanied by elevated ROS production under iron overload conditions induced by ferric ammonium citrate treatment. Knockdown of transferrin receptor 1, a key mediator of iron uptake, reduced these effects, suggesting that targeting iron homeostasis may represent a potential therapeutic strategy for PD (Shen et al. 2024).

##### 4.2.2.2 Mouse Models and Patients With PD and Neuronal Senescence Readouts

Expanding the research to animal models, the underlying mechanism of SATB1‐associated PD pathology was analyzed in a mouse model with downregulated SATB1 in midbrain neurons, using stereotactic adeno‐associated virus 1 injection expressing shRNA. Analysis of brain slices revealed loss of tyrosine hydroxylase (TH), lipofuscin accumulation, and upregulation of CDKN1A and SASP factors (e.g., CCL‐1) in surviving DA neurons. Importantly, consistent with previous in vitro findings, p16^INK4a^ was absent in both control and SATB1‐deficient DA. SATB1 knockdown also increased the number of activated microglia in the vicinity of TH‐positive neurons, indicating an accompanying neuroinflammatory response. In line with its clinical relevance, CDKN1A was markedly upregulated in dopaminergic neurons of the SNpc in post‐mortem PD brains compared with age‐matched controls, reinforcing the link between SATB1‐p21^Waf1/Cip1^‐mediated senescence and PD pathology (Riessland et al. 2019).

In vivo studies on C57Bl/6 mice injected with 5 μg of α‐syn PFFs and postmortem midbrain samples from PD patients reflected similar trends to the in vitro observations (Verma et al. 2021). Mice exhibited reduced Lamin B1 and p16^INK4a^ levels, alongside increased p21^Waf1/Cip1^ and decreased β‐III‐tubulin after 5–6 months, indicative of glial activation and potential neurodegeneration. Human PD samples revealed significantly lower Lamin B1 and SATB1, with elevated p21^Waf1/Cip1^ compared to controls, highlighting the potential role of senescence in PD pathology. However, the data suggest that, in this model, α‐syn PFF–induced senescence may arise predominantly in astrocytes and microglia, with neurons being affected more indirectly, underscoring the complex interplay between glial and neuronal compartments in the development and progression of PD (Verma et al. 2021).

Another study explored the effects of α‐syn‐A53T overexpression in the SNpc of C57BL/6 mice after recombinant AAV‐α‐syn‐A53T injection. The behavioral tests revealed that α‐syn‐A53T overexpression for 4 weeks decreased exploratory activity, impaired motor coordination, and decreased numbers of TH‐positive neurons, alongside reduced TH protein levels, suggesting loss of DA neurons and motor dysfunction. However, such effects were not observed at one or 2 weeks of overexpression. Particularly in 1 week of overexpression, a significant increase in SA‐β‐gal, γH2AX, p16^INK4a^, p21^Waf1/Cip1^, lipofuscin accumulation, mitochondrial dysfunction, oxidative stress, iron dysregulation, as well as elevated levels of pro‐inflammatory cytokines (e.g., IL‐4/5/6) and microglial activation, were observed. Treatment with iron chelator deferoxamine for 1 week before and continually for another week after AAV injection ameliorated the iron dysregulation, microglia activation, as well as the expression of p16^INK4a^ and p21^Waf1/Cip1^. Considering the results from in vitro experiments in PC12 cells, where stable overexpression of α‐syn‐A53T was sufficient to induce a senescence‐like phenotype, and the observation in transgenic mice that early α‐syn‐A53T overexpression does not yet cause dopaminergic neuron loss or overt motor dysfunction, cellular senescence may emerge before overt neurodegeneration in PD models. These findings raise the possibility that senescence, potentially also occurring in neurons, could represent an early pathogenic event that precedes and contributes to subsequent neuronal loss and clinical symptom onset. Intriguingly, targeting the iron dyshomeostasis driven by α‐syn‐A53T overexpression may open new therapeutic avenues to attenuate its toxic effects (Shen et al. 2024).

Collectively, these studies support a model in which α‐syn pathology, SATB1–p21^Waf1/Cip1^ signaling, iron dysregulation and mitochondrial dysfunction are recurrently associated with senescence‐like programs in dopaminergic neurons and glial cells, and can emerge before overt neuron loss and motor symptoms in PD models. At the same time, the senescence‐associated phenotypes and marker combinations reported across PD studies are highly variable, with glial senescence often more prominent than neuronal changes, making it difficult to determine whether neuronal senescence represents a primary driver of PD progression or a secondary stress response within a complex, cell type–specific neurodegenerative process.

Similar to observations in AD, this heterogeneity in marker composition and temporal dynamics highlights the challenge of distinguishing neuronal senescence from broader stress and neurodegenerative responses. The diversity of senescence‐associated phenotypes reported across neuronal models is summarized in Table 2.

### 4.3 Glial and Vascular Senescence in Brain Aging and Neurodegeneration

Although the primary focus of this review is neuronal senescence, accumulating evidence indicates that astrocytes, brain endothelial cells, and, to a lesser extent, microglia also acquire senescent or senescence‐like phenotypes during aging and neurodegenerative diseases. Across post‐mortem tissue and experimental models, senescent glial and endothelial cells co‐localize with misfolded protein aggregates, low‐grade chronic inflammation and progressive synaptic and neuronal loss, indicating that non‐neuronal senescent states actively influence the microenvironment in which neurodegeneration unfolds (Bhat et al. 2012; Baker and Petersen 2018).

Astrocytes are central components of the neurovascular unit, where they provide metabolic support, regulate neurotransmitter uptake, maintain ion and water homeostasis, and support the structural and functional integrity of the blood–brain barrier (BBB) (Sofroniew and Vinters 2010). Senescent astrocytes (astrosenescence) display several hallmarks of cellular senescence, including SA‐β‐gal activity, p16^INK4a^ and p21^Waf1/Cip1^ upregulation, DNA‐damage foci, Lamin B1 loss and chromatin reorganization together with a SASP enriched in IL‐6, IL‐8, IL‐1β and matrix metalloproteinases (MMPs) (Bhat et al. 2012; Bitto et al. 2010; Crowe et al. 2016). In AD, p16^INK4a^‐ and MMP‐1 positive senescent astrocytes accumulate in the cortex and hippocampus (Bhat et al. 2012). Experimental studies further demonstrate that Aβ and tau induce senescence‐like phenotypes in astrocytes, including acquisition of a SASP, both in vitro and in vivo (Shang et al. 2020). Consistent with a pathogenic role, selective ablation of p16^INK4a^‐positive glial cells in tau transgenic mice attenuates tau pathology and cognitive decline (Bussian et al. 2018). Evidence for astrosenescence in PD is converging from human tissue and toxin‐based models. Substantia nigra from PD patients harbors astrocytes with increased nuclear size, SA‐β‐gal, p16^INK4a^, IL‐6, IL‐1α, IL‐8 and MMP‐3, and reduced Lamin B1, consistent with a senescent astrocytic state (Chinta et al. 2013; Simmnacher et al. 2020). Paraquat exposure induces astrocyte senescence and a pro‐inflammatory SASP in vitro and in vivo, and targeted depletion of p16^INK4a^‐positive cells in paraquat‐treated mice preserves nigrostriatal DA neurons and improves motor performance, supporting a contributory role for astrocyte senescence and its SASP in DA neuronal vulnerability (Chinta et al. 2018).

Microglia also show aging and disease ‐associated transitions toward senescence‐like states, characterized by impaired phagocytosis, iron accumulation and a SASP‐like cytokine profile (Angelova and Brown 2019). However, current evidence more strongly supports the existence of microglial senescence‐like or chronically activated phenotypes that share several features of senescence without fulfilling all canonical hallmarks of the senescent state (Miller et al. 2022; Han et al. 2024).

Vascular senescence likewise emerges as an important contributor to brain aging and neurodegenerative pathology. Cerebrovascular endothelial cells are core elements of the neurovascular unit and BBB and are increasingly recognized as early targets of age‐associated vascular injury and senescence (Graves and Baker 2020; Csik et al. 2025). Senescent brain endothelial cells have been reported to exhibit cell‐cycle arrest, mitochondrial dysfunction, reduced nitric oxide bioavailability, increased endothelin and ROS, and downregulation or mislocalization of tight junction proteins. These alterations, together with the acquisition of a SASP, promote BBB leakage and neurovascular uncoupling (Yamazaki and Kanekiyo 2017; Hogans et al. 2025). In AD and PD, neurovascular unit dysfunction and BBB breakdown are now recognized as early pathophysiological events, and recent studies in mice demonstrate that senescent microvascular endothelial cells are key drivers of neurovascular coupling deficits and cognitive decline, whereas senolytic clearance of these cells restores BBB integrity, capillary density and learning performance (Csik et al. 2025; Liu et al. 2023).

Together, these observations underscore that brain aging and neurodegeneration involve senescence‐like states across multiple neural and vascular cell types. While the primary focus of the present review remains on neurons, the comparatively robust evidence for astrocytic and endothelial senescence emphasizes that neuronal phenotypes should be interpreted within the broader multicellular context of the neurovascular unit. This further highlights the importance of developing cell‐type‐specific frameworks for defining and interpreting senescence in the CNS.

### 4.4 Future Experimental Frameworks for Neuronal Senescence Research

Advances in peripheral tissues have been instrumental in defining the molecular hallmarks of senescence, but translating these concepts to the brain exposes substantial divergence in experimental systems, markers, and even the criteria used to classify cells as senescent. As illustrated in Table 2, neuronal studies frequently rely on distinct combinations of markers and often arrive at partially overlapping definitions of senescence. Peripheral studies have largely relied on proliferative cell types and robust cell‐cycle arrest readouts, whereas neuronal work spans rodent primary neurons, physiologically aged animals, human iPSC‐derived neurons, directly converted iNs, long‐term cultures, toxin‐based models, and human post‐mortem tissue, each with distinct advantages and limitations. This heterogeneity complicates direct comparison across studies and raises the risk that neuronal “senescence” is being defined in non‐equivalent ways.

Discrepancies in model systems are mirrored by inconsistencies in the markers used to label neurons as senescent. Classical senescence markers such as p16^INK4a^, p21^Waf1/Cip1^ and γH2AX are widely applied in brain studies, yet their specificity and reliability in post‐mitotic neurons are uncertain, given that neurons do not undergo classical cell‐cycle arrest and may engage distinct stress‐response and survival programs. Emerging single‐cell and single‐nucleus analyses of human post‐mortem cortex suggest that neuronal senescence‐associated signatures are mosaic, vary across cell types (e.g., glial or peripheral cells) (Herdy et al. 2024). These signatures might also differ between brain regions and disease stages and only partially overlap with canonical senescence signatures defined in peripheral proliferative cells. Overall, current evidence argues against a simple transfer of peripheral marker panels into the CNS and instead supports the need for neuron‐tailored criteria.

A key next step is therefore the systematic development of refined, neuron‐focused senescence marker sets. Such panels should be grounded in integrated experimental evidence from diverse in vitro and in vivo models and supported by convergent transcriptomic, epigenetic and functional readouts. Robust neuron‐specific senescence signatures would substantially improve the ability to track senescent neurons in cell culture, animal models and human tissue, and are a prerequisite for determining when and where neuronal senescence emerges, and whether it acts as a driver, amplifier or bystander in neurodegeneration.

Multi‐omic approaches provide a practical route toward this goal. Integration of single‐nucleus RNA‐seq with spatial transcriptomics and proteomics is beginning to map cell type‐specific aging and senescence trajectories and to identify candidate regulators that may be selectively engaged in disorders such as AD and PD. In parallel, aligning these human data with readouts from iPSC‐derived neurons, iNs, long‐term cultures and in vivo aging models should help distinguish processes that are truly driven by senescence from more general stress responses. Such an integrated, body‐to‐brain framework will be essential not only for mechanistic understanding, but also for the rational design of senolytic and SASP‐modulating interventions that are targeted to specific cell types, disease stages and vulnerable circuits rather than extrapolated wholesale from peripheral tissues.

Current evidence suggests that neuronal senescence is unlikely to represent a single, uniform role across neurodegenerative diseases, but rather a context‐dependent process that may act at different stages as a predisposing state, a disease‐promoting catalyst, or an aggravating factor. Building on earlier work linking cellular senescence to AD and PD (Kritsilis et al. 2018; Wang et al. 2024; Ma et al. 2025), recent studies and perspectives on neuronal senescence in the aged brain and neurodegeneration propose that stress‐induced, apoptosis‐resistant neurons with DNA damage, chromatin remodeling, and SASP output can accumulate and influence disease trajectories (de Luzy et al. 2024; Cantero‐Fortiz et al. 2026). In support of a predisposing or catalytic role, several models show that senescence‐like changes emerge before overt neuronal loss or robust clinical phenotypes, including early induction of p21^Waf1/Cip1^ and p16^INK4a^ (or p19), SA‐β‐gal activity, γH2AX foci, Lamin B1 loss, lipofuscin accumulation, mitochondrial dysfunction, and SASP‐related cytokine and chemokine changes in the context of Aβ, tau and α‐syn pathology (Ho et al. 2019; Verma et al. 2021; Yoon et al. 2022; Li et al. 2024; Du et al. 2024). Recent bioinformatic analyses of human brain tissue further identify neuron subpopulations with cell‐cycle re‐entry signatures, DNA damage responses, CDKN1A induction and curated senescence‐associated transcriptional programs that are more prevalent in neurodegenerative brains than in physiologically aged controls, consistent with an early, vulnerability‐shaping role (Herdy et al. 2024; Wu et al. 2024).

At the same time, synthesis across AD and PD models and reviews more strongly supports neuronal senescence as a disease‐modifying catalyst or aggravating factor than as a single initiating event. Senescent‐like neurons and glia have been linked to SASP production, gliosis, altered microglial and astrocytic reactivity, impaired neurogenesis, synaptic dysfunction, and resistance to apoptosis, all of which can amplify local proteinopathy and bystander damage (Baker and Petersen 2018; Kritsilis et al. 2018; Martinez‐Cue and Rueda 2020; Jurk et al. 2012; Cantero‐Fortiz et al. 2026). Importantly, recent reviews argue that the most reproducible readouts across neuronal senescence studies are not a single canonical marker, but multimarker combinations that include CDKN1A/p21^Waf1/Cip1^, CDKN2A/p16^INK4a^ or p19, γH2AX, Lamin B1 loss, lipofuscin accumulation, mitochondrial dysfunction, and context‐specific SASP factors such as IL‐6, CCL2, MCP‐1, MMPs and CXCL chemokines (Herdy et al. 2024; de Luzy et al. 2024; Cantero‐Fortiz et al. 2026; Campisi and d'Adda di Fagagna 2007). Yet, several authors caution that these markers are neither universal nor specific in post‐mitotic neurons and may partly reflect adaptive stress responses, aberrant cell‐cycle re‐entry, or glia‐driven inflammatory remodeling rather than a fully established senescent state (Kritsilis et al. 2018; Tan et al. 2014; Eitan et al. 2014; Ma and Farny 2023). Thus, the field currently favors a model in which neuronal senescence most plausibly contributes to disease progression by lowering stress resilience and amplifying pathogenic cascades, while definitive proof that it acts as a primary upstream driver of neurodegenerative disease onset remains limited and will require neuron‐centered, lineage‐tracing and senolytic intervention studies in vivo.

## 5 Summary

Cellular senescence has shifted from a peripheral, proliferative cell‐centric concept to one that also encompasses post‐mitotic neurons and glia. Work in neuronal cultures, aged animals, toxin‐based and transgenic models shows that neurons can acquire senescence‐like features including persistent DNA damage, chromatin remodeling, mitochondrial dysfunction and SASP‐like signaling, that intersect with key pathologies in AD and PD. At the same time, the field has largely imported markers and criteria from peripheral systems, leading to substantial variability in senescence readouts, model choice and thresholds for labelling cells as “senescent”, which complicates interpretation across studies.

This review underscores how heterogeneity in markers (e.g., p16^INK4a^, p21^Waf1/Cip1^, SASP components, DNA damage markers) and in experimental setups (iPSC‐derived neurons versus iNs, primary rodent neurons, long‐term cultures, toxin and transgenic models, human post‐mortem tissue) shapes current views on neuronal senescence. We argue that neuron‐adapted, multi‐parametric senescence signatures, supported by integrated molecular and functional data, are now needed to distinguish genuinely senescent cells from generically stressed neurons. As these detection strategies improve, they are likely to guide more precise targeting of senescent cells in the nervous system and to inform the design of senolytic and SASP‐modulating approaches for neurodegenerative disease and age‐related decline of nervous system functions.

Overall, the available data therefore support neuronal senescence most consistently as a context‐dependent catalyst and aggravating factor in neurodegeneration, with predisposing roles likely in specific settings where senescence‐like programs emerge before overt neuronal loss, but with only limited direct evidence that senescent neurons act as primary initiating events in AD or PD. Across the models and human studies considered, senescence‐associated phenotypes in neurons and glia have been identified using combinations of markers such as p21^Waf1/Cip1^, p16^INK4a^ or p19, γH2AX, SA‐β‐gal, Lamin B1 loss, lipofuscin accumulation, mitochondrial and lysosomal dysfunction, and diverse SASP components (including IL‐6, IL‐1 family cytokines, CCL2/MCP‐1, MMPs and chemokines). These patterns collectively underscore that multimarker panels and disease‐specific contexts are essential for interpreting neuronal senescence as a predisposing, driving or aggravating factor in neurodegenerative pathogenesis (Bussian et al. 2018; Kritsilis et al. 2018; Martinez‐Cue and Rueda 2020; Herdy et al. 2024; de Luzy et al. 2024; Cantero‐Fortiz et al. 2026).

## Author Contributions

K.M. prepared the original draft of the manuscript. M.U.D.M. contributed to manuscript editing, restructuring, rewriting, text refinement and revision. D.F. conceptualized the review, structure and scientific direction and writing, performed revision, proofreading, and editing of the final version.

## Funding

Funded by the EU NextGenerationEU through the Recovery and Resilience Plan for Slovakia under the project No. 09I01‐03‐V05‐00018.

## Conflicts of Interest

The authors declare no conflicts of interest.
